# A Proline-Hinge Alters the Characteristics of the Amphipathic α-helical AMPs

**DOI:** 10.1371/journal.pone.0067597

**Published:** 2013-07-23

**Authors:** Jong Kook Lee, Ramamourthy Gopal, Seong-Cheol Park, Hyun Sook Ko, Yangmee Kim, Kyung-Soo Hahm, Yoonkyung Park

**Affiliations:** 1 Research Center for Proteinaceous Materials (RCPM), Chosun University, Kwangju, Korea; 2 Department of Bioscience and Biotechnology, Bio/Molecular Informatics Center, Konkuk University, Seoul, Korea; 3 Department of Biotechnology, Chosun University, Kwangju, Korea; BioScience Project, United States of America

## Abstract

HP (2–20) is a 19-aa, amphipathic, α-helical peptide with antimicrobial properties that was derived from the N-terminus of *Helicobacter pylori* ribosomal protein L1. We previously showed that increasing the net hydrophobicity of HP (2–20) by substituting Trp for Gln^17^ and Asp^19^ (Anal 3) increased the peptide's antimicrobial activity. In hydrophobic medium, Anal 3 forms an amphipathic structure consisting of an N-terminal random coil region (residues 2–5) and an extended helical region (residues 6–20). To investigate the structure-activity relationship of Anal 3, we substituted Pro for Glu^9^ (Anal 3-Pro) and then examined the new peptide's three-dimensional structure, antimicrobial activity and mechanism of action. Anal 3-Pro had an α-helical structure in the presence of trifluoroethanol (TFE) and sodium dodecyl sulfate (SDS). NMR spectroscopic analysis of Anal 3-Pro's tertiary structure in SDS micelles confirmed that the kink potential introduced by Pro^10^ was responsible for the helix distortion. We also found that Anal 3-Pro exhibited about 4 times greater antimicrobial activity than Anal 3. Fluorescence activated flow cytometry and confocal fluorescence microscopy showed that incorporating a Pro-hinge into Anal 3 markedly reduced its membrane permeability so that it accumulated in the cytoplasm without remaining in the cell membrane. To investigate the translocation mechanism, we assessed its ability to release of FITC-dextran. The result showed Anal 3-Pro created a pore <1.8 nm in diameter, which is similar to buforin II. Notably, scanning electron microscopic observation of *Candida albicans* revealed that Anal 3-Pro and buforin II exert similar effects on cell membranes, whereas magainin 2 exerts a different, more damaging, effect. In addition, Anal 3-Pro assumed a helix-hinge-helix structure in the presence of biological membranes and formed micropores in both bacterial and fungal membranes, through which it entered the cytoplasm and tightly bound to DNA. These results indicate that the bending region of Anal 3- Pro peptide is prerequisite for effective antibiotic activity and may facilitate easy penetration of the lipid bilayers of the cell membrane.

## Introduction

Antimicrobial peptides have been classified into four major structural groups: α-helices, β-sheets, extended helices and loops. Of those, amphipathic α-helical structures are the most widely distributed among naturally occurring antimicrobial peptides [1]. The mechanisms of action for several, including defensin [2], cecropin [3], magainin [4], protegrin [5], melittin [6] and buforins [7], have been investigated, but the precise mechanism for their broad spectrum antimicrobial activity is still not fully understood. Evidence suggests, however, that they target the outer and inner bacterial membranes, ultimately either disrupting the cell membrane [2] or causing cooperative permeabilization [8].

When grown on plates, *Helicobacter pylori* produces cecropin-like antibacterial peptides to which it is, itself, resistant. These peptides have been attributed to fragments from the amino-terminus of ribosomal protein L1 (e.g., HP (2–20)) [9]. In a previous study, we designed a set of HP (2–20) analogues that varied only with respect to their net hydrophobicity [10]. Among them, Anal 3 showed much stronger antimicrobial activity against a broad spectrum of microorganisms than the others, most likely by targeting the bacterial membrane and killing via cell lysis. NMR spectroscopic analysis of the tertiary structure of Anal 3 in SDS micelles revealed that it has the longest α-helix structure among HP (2–20) analogues, which might account for its greater activity.

To better understand the structure-activity relationship of Anal 3, in the present study, we constructed an additional analogue by substituting the kink amino acid Pro for Glu^9^, thereby creating a helix-hinge-helix peptide (Anal 3-Pro). By analyzing circular dichroism (CD) spectra, we then determined the conformations of Anal 3-Pro in membrane-mimicking environments, after which NMR spectroscopy was used to determine the tertiary structure of the peptide. We then assayed the antimicrobial efficacy of these peptides against several bacteria and pathogenic fungal cells, and the cytotoxic activity against human red blood cells (RBCs). In addition, the mechanisms of action of Anal 3 and Anal 3-Pro were compared in *C. albicans*.

## Materials and Methods

### Peptide Preparation

The peptides were synthesized using the solid phase method with Fmoc(9-fluorenyl-methoxycarbonyl)-chemistry [11]. Peptide purification was then carried out using preparative HPLC on a C_18_ reverse-phase column. The amino acid compositions of the purified peptides were confirmed using an amino acid analyzer (HITACHI 8500A, Japan). The molecular weights of the synthetic peptides were determined using a matrix-assisted laser desorption ionization (MALDI) mass spectrometer.

### CD

To determine the secondary structure of the peptides in membrane-mimetic environments, CD experiments were carried out using a J720 spectropolarimeter (Tokyo, Japan) with a cell having a 1-mm path length. The peptide CD spectra in water, 50% TFE/water solution and 100 mM SDS micelles were recorded at 25°C at wavelengths ranging from 190 to 250 nm obtained at 0.1 nm intervals. The peptide concentration was 100 µM. For each spectrum, the data from four scans were averaged and smoothed using the J720/98 system program (Version 120C). CD data were expressed as mean residue ellipticity [θ] [12].

### NMR and structure calculation

Perdeuterated sodium dodecylsulfate (SDS-d_25_) was purchased from Cambridge Isotope Laboratories Inc, after which the conformation of the peptides in a membrane-mimicking environment (SDS micelles) was determined using NMR. The sample concentration was 1.0 mM in 0.45 ml of H_2_O/D_2_O (9∶1,v/v; pH 4.0) containing 150 mM SDS micelles. All phase-sensitive two-dimensional experiments (i.e., DQF-COSY, TOCSY and NOESY) were carried out using the time-proportional phase incrementation method [13], [14]. Structural calculations were carried out using X-PLOR version 3.851 [15] with the topology and parameter sets topallhdg and parallhdg, respectively. Standard pseudoatom corrections were applied to the non-stereospecifically assigned restraints [16], and an additional 0.5 Å was added to the upper bounds for NOEs involving methyl protons [17]. A hybrid distance geometry-dynamical simulated annealing protocol [18] was employed to generate the structures.

### Microbial strains

*Streptococcus aureus* (KCTC 1621), *Bacillus subtilis* (KCTC 1918), *Staphylococcus epidermidis* (KCTC 1917), *Escherichia coli* (KCTC 1682), *Salmonella typhimurium* (KCTC 1926), *Proteus vulgaris* (KCTC 2433), *Pseudomonas aeruginosa* (KCTC 1637), *Candida albicans* (KCTC 7270), *Saccharomyces cerevisiae* (KCTC 7296), and *Trichosporon beigelii* (KCTC 7707) were obtained from the Korean Collection for Type Cultures (KCTC), Korea Research Institute of Bioscience & Biotechnology (KRIBB), Taejon, Korea.

### Antimicrobial assays

The bacteria were grown to the mid-logarithmic phase in medium comprised of (in g/l) 10 bactotryptone, 5 yeast extract and 10 NaCl (pH 7.0). To assay antibacterial activity, each peptide was diluted stepwise (concentrations: 100, 50, 25, 12.5, 6.25, 3.125, 1.56, 0.78, 0.39, 0.195, 0.097 µM) in 1% bactopeptone medium. The tested organism (final bacterial suspension: 5×10^3^ colony formation units (CFU)/ml) was suspended in 100 µl in growth medium, mixed with 100 µl of test peptide solution, and seeded into the wells of a microtiter plate. Bacterial growth was then measured as an increase in optical density at 620 nm after incubating 10 h at 37°C.

To assay antifungal activity, cells were seeded into the wells of a flat-bottom 96-well microtiter plate (Greiner, Nurtingen, Germany) containing YPD (Dextrose 2%, Peptone 1%, Yeast extract 0.5%, pH 5.5) medium. The serially diluted-peptide solutions (50, 25, 12.5, 6.25, 3.125, 1.56, 0.78 and 0.39 µM) were then added, and the cell suspension was incubated for 24 h at 28°C. Thereafter, 10 µl of 3-(4, 5-dimethyl-2-thiazolyl)-2, 5-diphenyl-2H-tetrazolium bromide (MTT) solution [5 mg/ml MTT in phosphate-buffered saline (PBS), pH 7.4] were added to each well, and the plates were incubated for an additional 4 h at 37°C. The turbidity of each well was measured based on the absorbance at 570 nm using a microtiter ELISA reader (Molecular Devices Emax, California, USA). All assays of antimicrobial activity were carried out in triplicate [19].

### Ethics Statement

This study was approved by the institutional ethics committee, and all healthy donors provided written informed consent before treatment. We processed to the Ethical standards of the Institutional Ethics Committee of Chosun University and to the checklist for ethical consideration of cytotoxicity studies (https://www.cre.or.kr/article/policy/1382313). Human red blood cells (RBCs) were obtained from blood freshly collected from healthy donors at the Chosun University Hospital in Kwangju (Republic of Korea). Moreover, this study received ethics approval from the Institutional Ethics Committee of Chosun University. The authors of this article were blinded to all personal data from the donors, and all blood donors remained anonymous. All procedures were carried out according to rules provided by the Institutional Ethics Committee of Chosun University. Samples of blood were obtained from 5 healthy donors. The samples were immediately stored at 4°C until needed.

### Preparation of human red blood cells (RBCs)

Human RBCs were collected by centrifugation and washed three times with PBS (pH 7.0). The RBCs (100 µl) were suspended at 8% (v/v) in PBS and were plated into a 96-well plate. Peptide solution (100 µl) was added to each well, after which the plates were incubated for 1 h at 37°C and then centrifuged at 150×*g* for 5 min.

### Hemolytic activity assay

The hemolytic activities of the peptides were evaluated by determining the release of hemoglobin from an 8% suspension of fresh human RBCs. A 100-µl aliquot of the 8% RBC suspension was added to the wells of a 96-well plate, after which 100 µl of peptide solution in PBS (pH 7.0) was added to a final peptide concentration of 100 µM. Hemolysis was then measured based on absorbance at 414 nm using an Emax plate reader. As controls, no and complete hemolysis were determined in PBS and 0.1% Triton-X 100, respectively. The degree of hemolysis was calculated using the following equation:
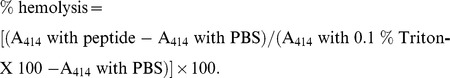



### Effect of the peptides on RBC morphology

RBCs were incubated for 1 h at 37°C with 60% of the minimal inhibitory concentration (MIC) of Anal 3-Pro or melittin. Negative controls were run without peptides. The RBCs were then fixed in 0.05 M cacodylate buffer (pH 7.2) containing equal volumes of 4% glutaraldehyde and 1% paraformaldehyde. After lyophilization and gold coating, the samples were examined on a HITACHI S-2400 (Tokyo, Japan).

### Flow cytometry

Membrane integrity following peptide treatment was evaluated using flow cytometry. Anal 3, Anal 3-Pro or melittin (control) was added to *C. albicans* cells (2×10^5^ cells in YPD media) to the MIC, after which cell membrane permeabilization was detected by incubating the treated cells in 50 µg/ml propidium iodide (PI) for 30 min at 4°C. After removing the unbound dye by extensively washing with PBS, the cells were subjected to flow cytometric analysis using a FACScalibur flow cytometer (Becton Dickinson, San Jose, CA). PI fluorescence was monitored in the FL2-H channel.

### Confocal laser-scanning microscopy

Intracellular localization of fluorescein isothiocyanate (FITC)-conjugated Anal 3, Anal 3-Pro or magainin II in C. alb*icans* was determined by confocal laser scanning microscopy. The cells were inoculated into 3 ml of yeast medium and then incubated for 12 h at 28°C. The FITC-labeled peptides were added to 100 µl of the cell suspension to the MIC, after which they cells were incubated for 15 min at 28°C. Intracellular localization of FITC-labeled peptides was determined using an Olympus IX 70 upright microscope (Olympus, Japan) equipped with a Leica TCS 4D laser-scanning system.

### Scanning electron microscopy (SEM)

Midlog phase *C. albicans* were resuspended at 10^8^ CFU/ml in Na-phosphate buffer (pH 7.4) supplemented with 100 mM NaCl (buffer A) and then incubated at 37°C with Anal 3-pro or magainin II. Controls were run in the absence of peptide solvent. After 30 min, the cells were fixed for 2 h at 4°C in 0.2 M Na-cacodylate buffer (pH 7.4) containing an equal volume of 5% glutaraldehyde. After fixation, the samples were filtered on Isopore filters (0.2 µm pore size, Millipore, Bedford, MA, USA) and extensively washed with 0.1 M Na-cacodylate buffer (pH 7.4). The filters were then treated with 1% osmium tetroxide, washed with 5% sucrose in cacodylate buffer and dehydrated in a graded ethanol series. After lyophilization and gold coating, the samples were examined on a HITHACHI S-2400 instrument (HITHACHI, Japan).

### Preparation of small unilamellar vesicles (SUVs)

SUVs were prepared by drying of PC/cholesterol (10∶1 w/w) under nitrogen, suspending the film by vortex mixing in buffer (50 mM phosphate buffer containing 100 mM, pH 7.5) and sonicating the suspension using a tip ultrasonicator. A drop of vesicles was deposited on a carbon-coated grid and negatively stained with 2% uranyl acetate. Specimens were examined in the TECNAI 12 at an accelerating voltage of 120 kV.

### Binding assay

Chitin (β-1,4-*N*-acetyl-D-glucosamine), chitosan (β-1,4-D-glucosamine), cellulose (β-1,4-glucan), curdlan (β-1,3-glucan) and peptidoglycan (β-1,4-glycosidic linkage between *N*-acetyl muramic and *N*-acetylglucosamine) were used for the *in vitro* binding assay of Anal 3-Pro. One hundred µg of each insoluble polysaccharide was added in 5 µg of purified Anal 3-Pro and incubated at room temperature with mild agitation for 1 h. The mixture was centrifuged (10,000×*g* for 2 min) and the pellet was washed three times with 1 ml of washing buffer (10 mM Tris, pH 7.5, 500 mM NaCl, 0.02% Tween 20). The peptide bound to insoluble polysaccaharide was detached by adding SDS-polyacrylamide gel electrophoresis sample buffer.

### DNA gel retardation

Plasmid pBluescript SK(+) (Stratagene) was purified using a plasmid extraction kit (Exprep™ Quick, GeneAll Biotechnology Co., Seoul, Korea). The plasmid DNA (200 ng) was then mixed with increasing amounts of peptides in 10 mM Tris buffer (pH 8.0) containing 1 mM EDTA, 5% glycerol, 20 mM KCl and 50 µg/ml BSA. The mixtures were incubated for 10 min at 37°C and then electrophoresed on a 0.5% agarose gel in the TBE buffer, after which the gels were stained with ethidium bromide [20]. Gel retardation was visualized under UV illumination using a Bio-Rad Gel Documentation system.

### Preparation of dextran-loaded liposomes and leakage experiments

FITC-labeled dextrans (FD-4, 10, 20, 40, 70 and 500) were utilized as model cytoplasmic components. FD (fluorescein isothiocyanate dextrans), with average molecular masses of 4, 10, 20, 40, 70, and 500 kDa were all purchased from the Sigma Chemical Co. (St. Louis, MO). FD-entrapping liposomes (unilamellar vesicles) with different lipid compositions were prepared using the reverse-phase evaporation method [21], after which the concentrations of the FD-entrapping vesicles were determined in triplicate using a phosphorus assay [22]. To prepare FD-entrapping liposomes, a buffer solution (buffer II: PBS) containing 2 mg/ml of the FD was sonicated for 30 min with a lipid solution in chloroform (20 mg/ml) on ice. The chloroform was then gradually removed using a rotary vacuum evaporator at 25°C, resulting first in the formation of a viscous gel and then a liposome suspension. Buffer (2 ml) was added, and the suspension was evaporated further to remove the remaining solvent. The liposome suspensions were then centrifuged and washed for several cycles at 22,000×g_av_ for 30 min in order to remove unentrapped-FD. The washed liposomes were extruded 30 times through polycarbonate filters (two stacked 0.4-µm pore size filters) using an *Avanti* Mini-Extruder (Avanti Polar Lipids inc., Alabaster, AL) to obtain liposomes of homogeneous size (∼400 nm). Aliquots of the peptide solutions at appropriate concentrations were incubated with a suspension of FD-loaded liposomes (100 µM) for 1–20 min at 25°C, and were then centrifuged for 30 min at 22,000×g. The supernatants were recovered and the leakage was recorded by monitoring the FITC fluorescence intensity (excitation wavelength: 494 nm, emission wavelength: 520 nm). 100% leakage was achieved by addition of Triton X-100 to a final concentration of 1 mM. The percent leakage value was then plotted.

## Results and Discussion

### Design and Peptide Preparation

HP (2–20) was previously shown to inhibit the growth of both bacteria and fungi [9], [23]. In an earlier study, we used an α-helical wheel diagram to show that HP (2–20) (AKKVFKRLEKLFSKIQNDK-NH_2_) is comprised of both hydrophobic and hydrophilic regions [8], and that substituting a Trp for the more hydrophilic amino acids Gln^17^ and Asp^19^ (Anal 3; AKKVFKRLEKLFSKIWNWK-NH_2_) caused a dramatic increase in antimicrobial activity. In the present study, we constructed an additional analogue, Anal 3-Pro, which contains a Glu^9^→Pro substitution (Anal 3-Pro; AKKVFKRLPKLFSKIWNWK-NH_2_), to investigate the contribution made by each structural element to the highly efficacious antimicrobial activity of Anal 3.

We selected Glu^9^→Pro substitution because the resultant peptide is negatively charged and does not favour electrostatic interaction between itself and the negatively charged bacterial membrane. Moreover, we wanted to introduce a hinge region into the middle of the peptide, and with the exception of Glu, all of the amino acids in that region of the peptide are essential for its antimicrobial activity. This mutation changed the peptide's conformation because proline is a potent α-helix breaker that has been found in the transmembrane helices of a variety of integral membrane proteins [24] and in a number of α-helical antimicrobial peptides that promote ion channel activity, including melittin [25], cecropin A [26], paradoxin [27], brevinins [28], gaegurin [29] and buforin II [8]. The primary structures of these cell-selective antimicrobial peptides consist of a hinge (a Pro residue) conecting N- and C-terminal α-helical segments, and two-dimensional NMR studies have shown that these peptides do indeed assume a helix-hinge-helix structure in membrane-mimicking environments [30]. The importance of the Pro residue was confirmed by the findings that Pro^14^→Ala substitution in the active analogue derived from gaegurin induced a significant reduction in antibacterial activity [31], while elimination of Pro^7^ from pardaxin increased hemolytic activity [27].

The effect of incorporating a Pro-hinge into amphipathic α-helical peptides that lack cell selectivity had not been investigated until now, however. We therefore introduced a Glu^9^→Pro substitution into HP (2–20) Anal 3 with the idea of creating a helix-hinge-helix peptide with no cell selectively (Anal 3-Pro). We then investigated the effect of the Pro incorporation on the α-helical structure of Anal 3 using CD and NMR studies, and compared the antimicrobial and hemolytic activities of Anal 3-Pro with those of the previously described HP (2–20) Anals 1–5 [10].

### CD Spectra

We used SDS micelles to examine the behaviour of the AMPs in a membrane-mimicking environment, and we used TFE as a co-solvent in CD spectroscopy studies of peptide folding. This solvent is often used to increase the propensity of AMPs to form secondary structure. We first investigated the secondary structures of HP (2–20), Anal 3 and Anal 3-Pro by examining their CD spectra in aqueous buffer, 50% TFE/water solution and SDS micelles. We found that the CD spectra for all three peptides showed a random coil structure in water (Figure S1) but an α-helical structure in a membrane-mimicking environment, such as 50% TFE/water solution or 150 mM SDS micelles. Moreover, the data indicate that Anal 3 has a more α-helical structure than Anal 3-Pro, suggesting the proline residue in the central region caused distortion of the helix.

### Resonance Assignment

Sequence-specific resonance assignment was carried out using DQF-COSY, TOCSY and NOESY data. Figure S2 and S3 show the NOESY spectra with sequential assignments of Anal 3-Pro in the HN-HN region. NOESY and TOCSY experiments carried out at 313K, 318K and 323K enabled complete assignment of the overlapping peaks. Chemical shifts within Anal 3-Pro in SDS micelles at 313K and pH 4.0 (relative to DSS) are listed in Table S1. Anal 3-Pro has a short helix extending from Lys^3^ to Leu^8^ and a long helix extending from Pro^10^ to Trp^19^ and a hinge region between the helices. The observed values of the ^3^J_HNα_ coupling constant for the helical region in the C-terminus of the peptide were generally less then 6 Hz. The values of the amide proton temperature coefficient were used to predict hydrogen bond donors; values more positive than −4.5 ppb/K were taken as indicating that the amide proton is involved in intramolecular hydrogen bonding.

### Tertiary Structures

The tertiary structures of Anal 3-Pro were determined using sequential (|*i−j*| = 1) and medium-range (1<|*i*−*j*|≤5) as well as restraints on intraresidual distances and torsion angles (Table S2). From among the structures, which were accepted with small deviations from the idealized covalent geometry and experimental restraints (≤0.05 Å for bonds, ≤5° for angles, ≤5° for chirality, ≤0.3 Å for NOE restraints and ≤3° for torsion angle restraints), the 20 output structures with the lowest energy were analyzed. The statistics for these 20 simulated annealing (SA) structures of Anal 3-Pro are given in Table S2. All displayed good covalent geometries and small NMR constraint violations. When we superimposed the 20 structures on the backbone atoms of the residues extending from Pro^10^ to Trp^19^, the rmsd from the mean structure was 0.77 Å for the backbone atoms (N, Cα, C′, O) and 1.46 Å for all heavy atoms. However, the rmsd from the mean structure for all residues was much larger for all peptides because of the kink in the peptide backbone if the region in the middle of the molecule.

**Figure 1:**
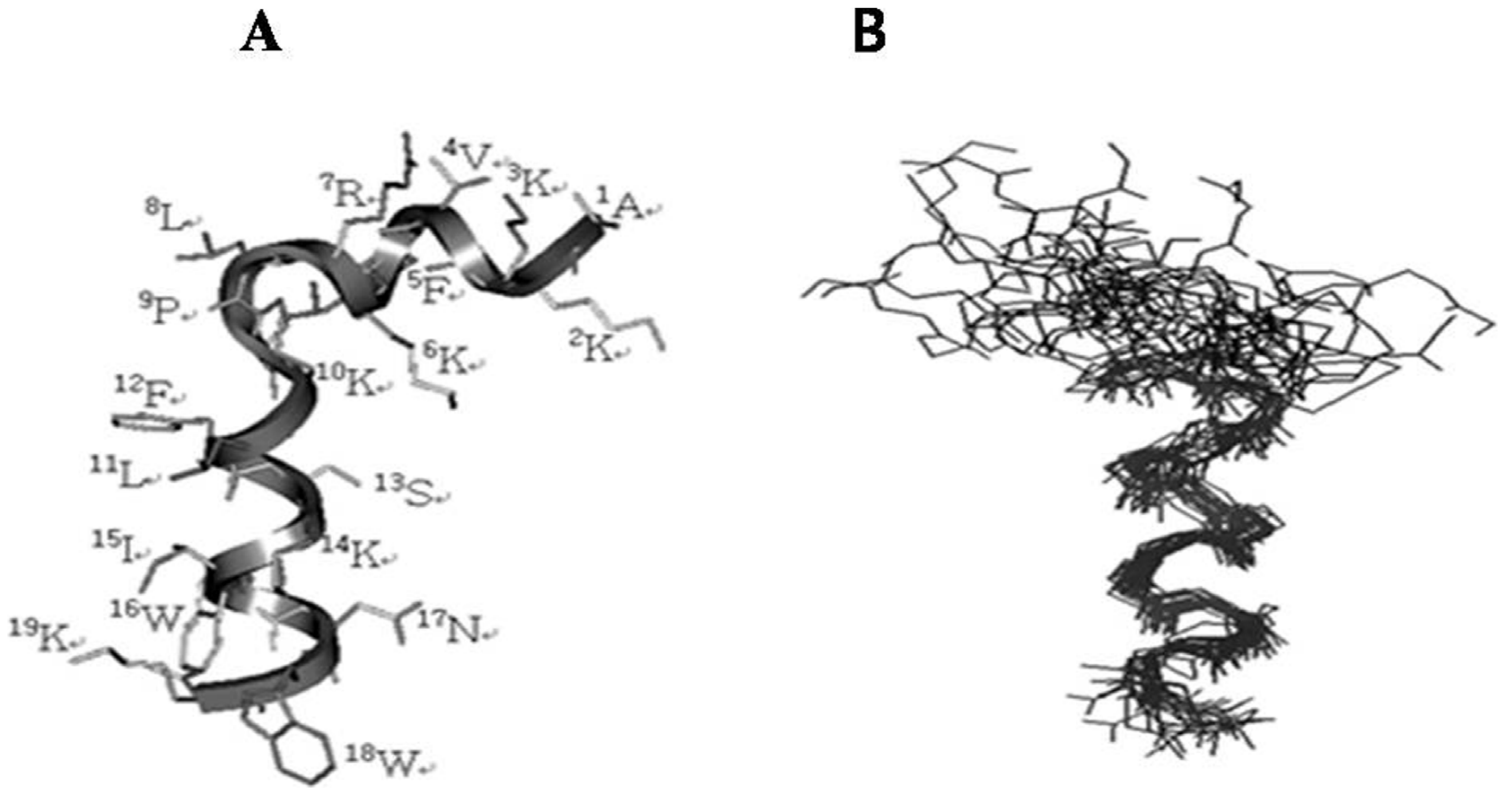
Tertiary structures. (A) Ribbon diagram of the lowest energy structure of Anal 3-Pro. (B) Superpositions of the 20 lowest energy structures calculated from the NMR data using the backbone atoms of residues 9–18.

**Figure 2:**
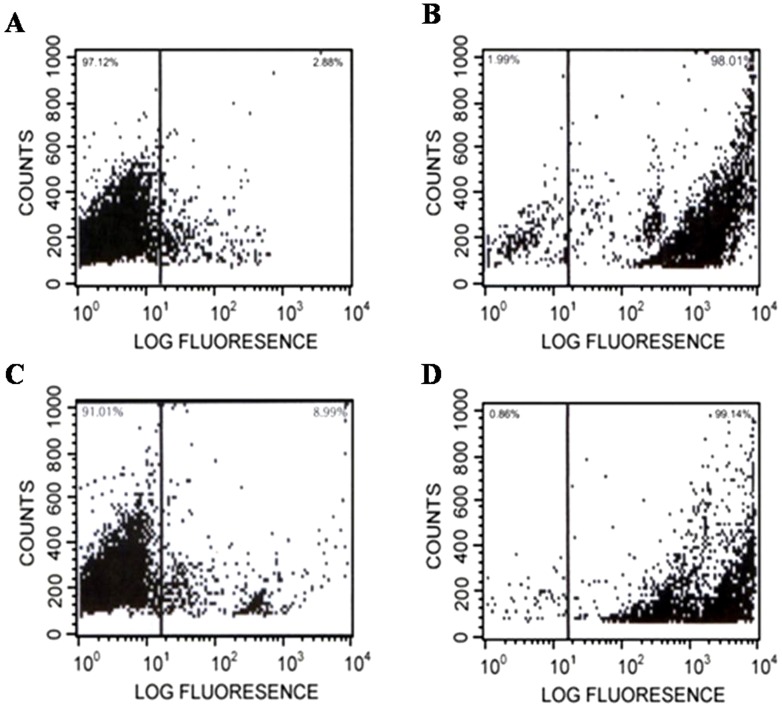
Flow cytometric analysis of cell membrane permeability. Flow cytometric analysis showing PI uptake by *C. albicans* cells treated with the MIC of Anal 3 or Anal 3-Pro. Increments in the log fluorescence signal represent peptide-induced PI uptake: (*A*) no peptide, (*B*) Anal 3, (*C*) Anal 3-Pro and (*D*) melittin.

Figure 1A shows a ribbon diagram of the lowest energy Anal 3-Pro structure, while in Figure 2B all heavy atoms in the region extending from Pro^10^ to Trp^19^ were superimposed with respect to the restraint-minimized average structure for all peptides. Using PROCHECK analysis, we determined that Anal 3-Pro contains a short α-helix, extending from Lys^4^ to Leu^9^ in the N-terminal region, and a long α-helix, extending from Pro^10^ to Trp^19^ in the C-terminal region, which are connected via the Pro^10^ residue.

### Antimicrobial and Hemolytic Activities

The antimicrobial activities of the peptides were evaluated *in vitro* against a representative set of bacterial strains, including three Gram-positive and four Gram-negative species, using the broth microdilution method. The respective MICs are summarized in Table 1. The data indicated that Anal 3-Pro had 4 times greater antibacterial activity than Anal 3 against all bacterial strains tested. In addition, when the MICs for the antifungal activity of the peptides were determined in MTT assays, we found that Anal 3-Pro had approximately twice the antifungal activity of Anal 3 against the microorganisms tested (Table 1). In particular, replacing Glu with Pro in the middle of the peptide sequence introduces a kink into the helix structure, which apparently increased the antimicrobial activity of Anal 3-Pro, as compared to Anal 3.

**Table 1:** Sequences, Biological activities of HP (2–20) and its analogues.

		MIC (µM)									
Name	Sequence	G(+)			G(−)				Yeast		
		*BS*	*SE*	*SA*	*EC*	*PV*	*ST*	*PA*	*CA*	*TB*	*SC*
**HP (2–20)**	AKKVFKRLEKLFSKIQNDK-NH_2_	1.56	3.13	12.5	6.25	3.13	0.78	12.5	25	12.5–25	25
**Anal 1**	AKKVFKRLEKLFSKIQNWK-NH_2_	1.56	0.78	3.13	1.56	1.56	0.39	6.25	12.5	6.25	12.5
**Anal 2**	AKKVFKRLEKLFSKIWNDK-NH_2_	1.56	1.56	3.13	1.56	1.56	0.78	6.25	12.5	6.25–12.5	12.5
**Anal 3**	AKKVFKRLEKLFSKIWNWK-NH_2_	0.78	0.78	1.56	0.78	1.56	0.39	3.13	6.25	3.13	3.13–6.25
**Anal 4**	AKKVFKRLEKSFSKIQNDK-NH_2_	6.25	12.5	>12.5	12.5	6.25	1.56	>12.5	50–100	50	50
**Anal 5**	AKKVSKRLEKLFSKIQNDK-NH_2_	3.13	6.25	>12.5	6.25	3.13	3.13	>12.5	100	50	50–100
**Anal 3-Pro**	AKKVFKRLPKLFSKIWNWK-NH_2_	0.39	0.39	0.39	0.19	0.09	0.09	1.56	3.13	1.56	1.56

G(+), Gram positive bacteria; G(−), Gram negative bacteria; *BS*, *B. substilis*; *SE*, *S. epidermides*; *SA*, *S. aureus*; *EC*,*E. coli*; PV, *P. vulgalis*; *ST*, *S. typhimurium*; *PA*, *P. aeruginosa*; *CA*, *C. albicans*; *TB*, *T. beigellii*; *SC*, *S. cerevisiae*.

We also evaluated the hemolytic activity of the peptides. Notably, none of the synthetic peptides tested showed any hemolytic activity. Anal 3-Pro, for example, exhibited no hemolytic activity at concentrations as high as ∼100 µM, despite its strong antimicrobial activity (Figure S4). Scanning electron microscopic examination of RBCs confirmed that, even at 100 µM, Anal 3-Pro elicited no change in RBC morphology and caused no damage to the cell membrane (compare Figure S4A and B). By contrast, at a concentration inducing 60% hemolysis, melittin, an antimicrobial peptide known to target the cell membrane [6], had a significant effect on RBC morphology, frequently inducing formation of pores in the membrane (Figure S4C). Thus, Anal 3-Pro shows remarkable antibacterial and antifungal activity with no hemolytic activity. Its further study may therefore not only increase our understanding of the mechanism of action of Anal 3-Pro and the mechanism underlying its cell-selectivity, but also facilitate the design of novel antibiotic peptide with improved antimicrobial activity and no cytotoxicity.

### Flow Cytometric Analysis of Cell Membrane Permeability

The precise mechanism of action of helix-hinge-helix antimicrobial peptides is not yet fully understood, this finding is consistent with the idea that disruption of the cell structure through pore formation [32] and/or ion channel generation [33] is the most likely mechanism. To investigate the extent to which Anal 3-Pro acts by damaging the plasma membrane or by affecting cell physiology, *C. albicans* were incubated with propidium iodide (PI) [32] plus Anal 3, Anal 3-Pro or melittin, after which intracellular PI fluorescence within individual cells was analyzed by FACScalibur flow cytometry.

Figure 2 shows a plot of forward light scatter (x-axis) against PI fluorescence (y-axis) in which each dot represents an individual cell. The results showed that whereas most cells left untreated (Figure 2A) or treated with Anal 3-Pro (Figure 2C) showed little or no PI signal, cells treated for 30 min on ice with 12.5 µM Anal 3 or melittin showed a dramatic rightward shift of the peak, indicating a large influx of PI (Figure 2, B and D). We found that when the MICs of peptides (Ana 3 and melittin) were applied to the pathogenic fungus *C. albicans*, they exhibited a strong capacity to permeabilize the cell membrane. By contrast, Anal 3-Pro caused only a very small amount of dye influx, suggesting a different mechanism of antimicrobial activity.

### Confocal Laser-Scanning Microscopy

**Figure 3:**
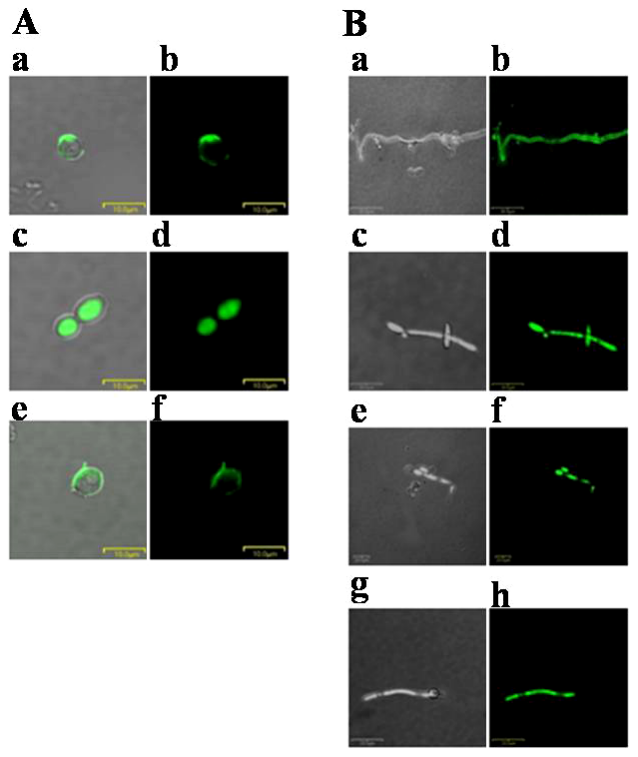
Confocal laser-scanning microscopy. Bright field (*Aa*, *Ac* and *Ae*) and confocal fluorescence (*Ab*, *Ad* and *Af*) micrographs of *C. albicans* cells treated with FITC-conjugated Anal 3 (*Aa* and *Ab*), Anal 3-Pro (*Ac* and *Ad*) or magainin II (*Ae* and *Af*). Bright field (*Ba*, *Bc*, *Be* and *Bg*) and confocal fluorescence (*Bb*, *Bd*, *Bf* and *Bh*) micrographs of *T. begellii* cells treated with FITC-conjugated Anal 3 (*Ba* and *Bb*), Anal 3-Pro (*Bc* and *Bd*), buforin II (*Be* and *Bf*) or TAT (*Bg* and *Bh*). Cells were incubated for 15 min at 28°C with the respective peptides. Note that whereas FITC-Anal 3-Pro, buforin II and TAT are distributed relatively uniformly in the cytoplasm, FITC-Anal 3 and FITC-Magainin II are localized to the periphery, presumably associated with the cell membrane.

The observed differences in the effects of Anal 3, Anal 3-Pro, buforin II and TAT on membrane permeability prompted us to investigate the site of action of Anal 3-Pro. To that end, *C. albicans* or *T. begellii* were incubated with FITC-conjugated Anal 3; Anal 3-Pro; magainin II, another antimicrobial peptide that targets the cell membrane; buforin II, an antimicrobial peptides that targets the cytoplasm after permeating a membrane micropore; or TAT, another antimicrobial peptide that targets the cytoplasm, after which the cells were visualized using confocal laser-scanning microscopy. We found that FITC-Anal 3 remained associated with the cell membrane (Figure 3, Aa, Ab, Ba and Bb), as did FITC-magainin II (Figure 3, Ae and Af). By contrast, Anal 3-Pro (Figure 3, Ac, Ad, Bc and Bd), buforin II (Figure 3, Be and Bf) and TAT (Figure 3, Bg and Bh) were distributed throughout the fungal cells, most likely targeting one or more cytoplasmic components or nucleic acids. Similar distributions of FITC-Anal 3-Pro were observed in *B. subtilis* and *E. coli* (data not shown).

### Non-morphological Changes Induced by Anal 3-Pro

Scanning electron microscopic examination of *C. albicans* treated with the respective peptides revealed that untreated cells had a normal, smooth surface (Figure S5A), that cells treated with Anal 3-Pro or buforin II had similarly smooth membranes (Figure S5B and D) and that cells treated with magainin II had a rough cell surface (Figure S5 *C*). This suggests that substituting Pro for Glu significantly reduced the degree of peptide-induced membrane disruption, as compared to Anal 3 [34].

### Effect of the Peptide on the Morphology of PC/Cholesterol SUVs Examined using Electron Microscopy

To confirm the ability of Anal 3-Pro to disrupt microbial cells, we carried out a set of experiments using liposomes. Artificial small unilamellar vesicles (SUVs) (phosphatidylcholine (PC)/cholesterol (CH); 10∶1, w/w) and neutral PC vesicles were used as model membrane systems. The antimicrobial activity of Anal 3 is based on the formation of transmembrane channels. SUVs were disrupted after treatment with Anal 3 (Figure S6B), suggesting the peptides perturb the lipid components of the plasma membranes. But, SUVs treated with Anal 3-pro were not disrupted and retained their vesicluar form (Figure S6C). This clearly indicates that the change in overall length caused by the helix distortion likely prevents favourable interactions across the membrane, such as those seen with the extended Anal 3 helix.

### Anal 3-Pro Recognizes Chitin and Peptidoglycan

**Figure 4:**
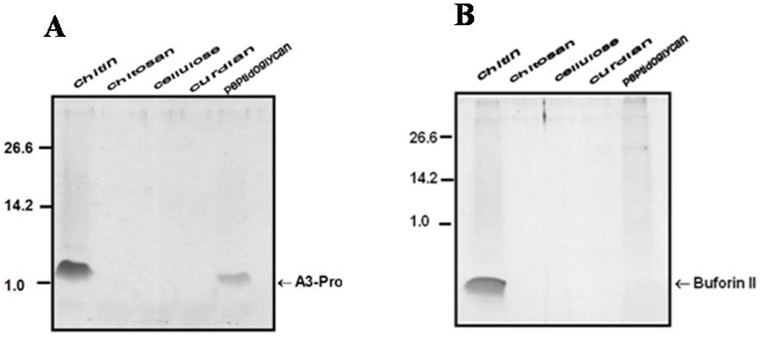
Anal 3-Pro recognizes chitin and peptidoglycan. One hundred µg of each insoluble polysaccharide was added in 5 µg of purified Anal 3-Pro and incubated at room temperature for 1 h with mild agitation. The peptide bound on insoluble polysaccaharide was detached by adding SDS-polyacrylamide gel electrophoresis sample buffer. The molecular mass makers are indicated in kDa.

The pattern motifs of the microbial cell wall components from gram-negative bacteria, gram-positive bacteria and yeast can initiate innate immune signaling. To examine the specificity of Anal 3-Pro binding, we incubated the cell wall components with the indicated peptide as described in the “Materials and Methods.” Using these binding assays, we specifically detected Anal 3-Pro in extracts containing chitin and peptidoglycan, whereas no binding activity was observed with chitosan, cellulose or β-1,3-glucan (Figure 4). Thus, Anal 3-Pro appears to recognize chitin and peptidoglycan and to permeabilize the cellular target site. By contrast, buforin II recognized only chitin.

### DNA Gel Retardation

To confirm the cellular target site of Anal 3-Pro, we also assessed retardation of DNA band migration. Given the concentration-dependent translocation of Anal 3-Pro into the cytoplasm of bacterial cells shown in Figure S7, we examined the binding to intracellular DNA. Anal 3-Pro was mixed with a fixed amount of plasmid DNA (pDNA, 100 ng), after which the complexes were electrophoresed on agarose gels. Retardation of the pDNA band was detected for a 600 ng peptide, indicating aggregation of the pDNA with Anal 3-Pro (Figure S7B). Additional in vitro experiments will be needed to determine how the peptide-DNA interaction is mediated. On the other hand, no band retardation was seen when pDNA was mixed with Anal 3 (Figure S7A), indicating pDNA and Anal 3 do not interact. In addition, buforin II (Figure S7C) and TAT (Figure S7D) were shown to induce retartation at different peptide concentrations, 1 µg or 400 ng, respectively.

### Sizes of the Different Pores Formed between Peptides

**Figure 5:**
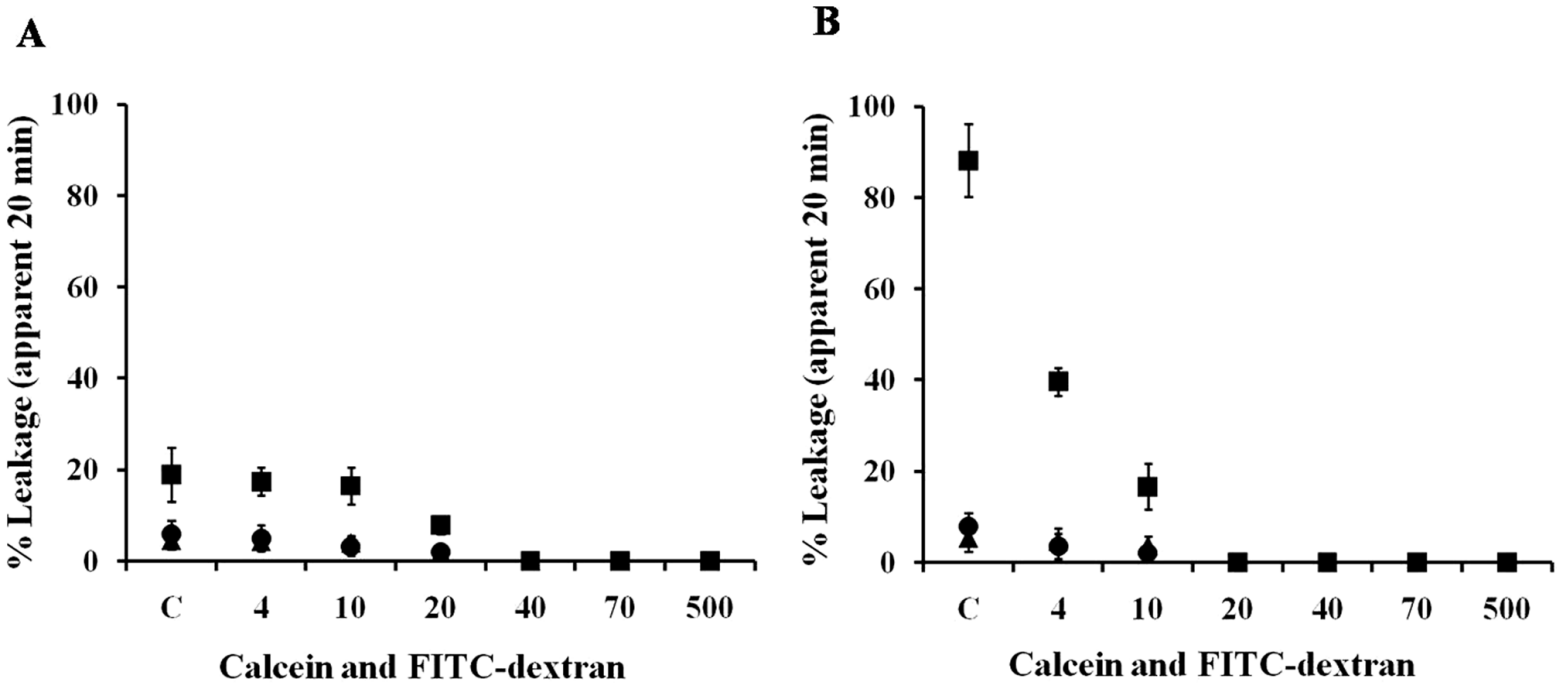
Sizes of the different pores formed between peptides. The lipid compositions (A, PE/PG (1∶1); B, PC/CH (1∶1)) of the liposomes containing calcein or FD4, 10, 20, 40, 70 or 500 are indicated in the text; they were prepared and quantified as described in Materials and Methods. The release of calcein and dextran were fluorometrically determined. The apparent percentage of release was calculated as 100×(*F-F_o_*)/(*F_t_-F_o_*), where *F* and *F_t_* are the fluorescence intensities prior to and after the addition of each peptide (▪, Anal 3-Pro; ▴, TAT; •, buforin II), respectively, and *F_o_* is the fluorescence of the intact vesicles. C represents calcein in each figure. Values are expressed as an average of three independent measurements. Error bars represent the standard error of the mean (n = 4).

To gather additional clues as to the type and extent of membrane damage induced by Anal 3-Pro, TAT and buforin II, we assessed the peptide-induced release of fluorescently labeled dextran molecules of various sizes – i.e., FD4 (3.9 kDa, 1.8 nm radius), FD10 (9.9 kDa), FD20 (19.8 kDa, 3.3 nm radius), FD40 (40.5 kDa, 4.8 nm radius), FD70 (71.6 kDa, 5 nm radius) and FD500 (530 kDa). Using FD-containing liposomes incubated with 10 µM peptide (peptide/lipid ratio = 1/10) [35], [36], we determined that the evoked release of FD varied inversely with its molecular mass/size (Figure 4A and B). Anal 3-Pro released barely 20% of FD4 from phosphatidylethanolamine (PE)/phosphatidyglycerol (PG) liposomes, while TAT and buforin II released only 5% of the FD4, and were unable to release any of the larger markers. For instance, Anal 3-Pro released 39.63% of the FD4 from PC/cholesterol (CH) liposomes, but only 16.65% of the FD10. TAT and buforin II respectively released only 4.7% and 3.6% of the FD4 from PC/CH vesicles, and were unable to release any of the larger markers. This indicates that the pore created by Anal 3-Pro has an estimated radius less than 1.8 nm, which is similar to pores induced by buforin II or TAT at 1/10 (peptide/lipid) molar ratio concentration (Figure 5A). When we then compared the release from PE/PG liposomes with that from PC/CH liposomes (Figure 5B), we determined that release from the former was peptide-specific, whereas release from the latter did not vary among the peptides.

**Figure 6:**
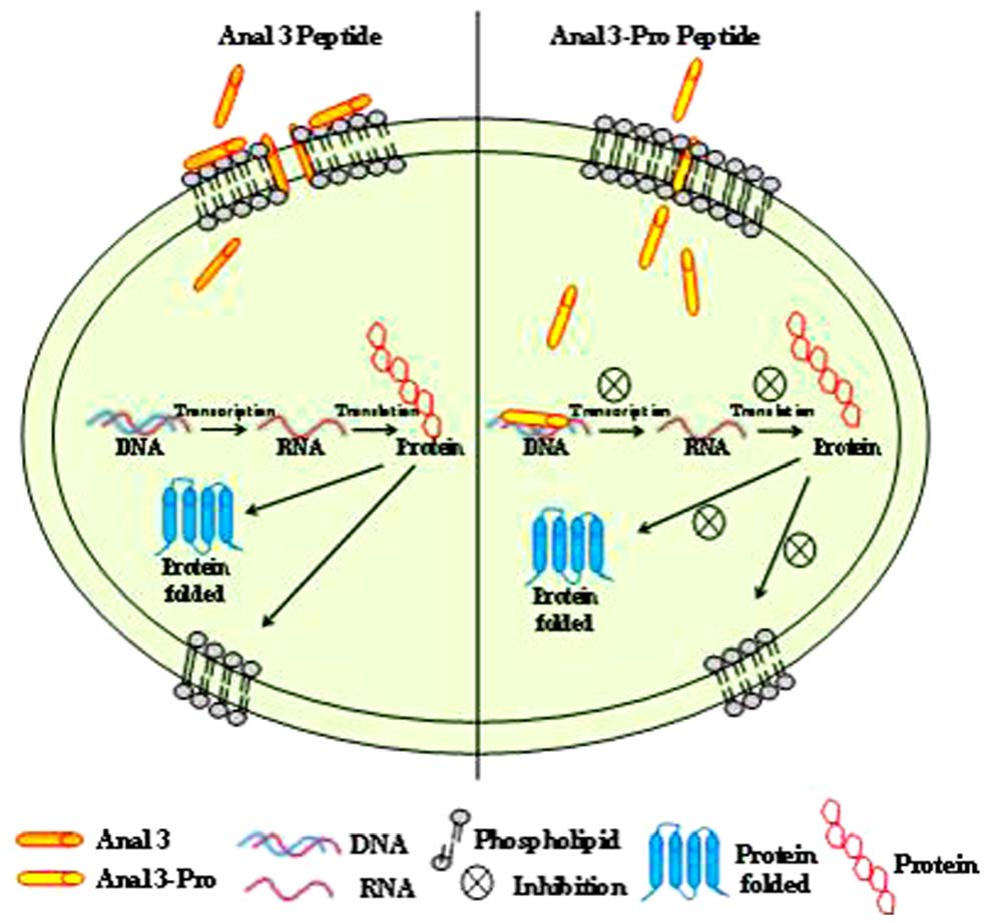
Modes of action of Anal 3 (A) and Anal 3-Pro (B). Schematic representation of the proposed mechanisms of action of Anal 3 and Anal 3-Pro in microbial cells.

In summary, to investigate the structure and mechanism of action of Pro-kink incorporation, we designed Anal 3-Pro in which the Glu at position 9 of Anal 3 was substituted with Pro introduced kink into α-helices. Anal 3-Pro exhibited an increase in antibiotic activity without a hemolytic effect. It appears that incorporating Pro into Anal 3 induces a more selective cytotoxicity against microorganisms with a change in the mode of action of the amphipathic α-helical peptide. The modes of action of Anal 3 and Anal 3-Pro are summarized in Figure 6. Anal 3 exerts its bactericidal effects by disrupting the membrane and inducing pore formation. By contrast, Anal 3-Pro acts by inducing formation of a short-lived micropore, permeating into the cytoplasm and then binding to the DNA. Our results clearly demonstrate that Anal 3-Pro constitutes a new class of antimicrobial peptide that targets intracellular components, most likely DNA, after permeating short-lived micropores in the cell membrane, and that the proline hinge is a key structural factor for the cell penetration. Thus insertion of a single amino acid to form a proline-hinge region can change a membrane-targeted peptide to a cell-penetrating one. This finding may provide an important clue to the design of future therapeutic antibiotic drugs, given its efficacy toward bacterial and fungal cells and its lack of toxicity toward eukaryotic cells such as human erythrocytes.

## Supporting Information

Figure S1 **CD spectra for HP (2–20) (○), Anal 3 (▾) and Anal 3-Pro (•) in water (A) 50% (v/v) TFE (B) or 100 mM SDS micelles in 10 mM sodium phosphate buffer (pH 7.2) (C).** In each case, the peptide concentration was 50 µM.
(TIF)

Figure S2 **NH-NH region of the NOESY spectrum of Anal 3-Pro in 150 mM SDS micelles (mixing time, 350 ms; 318K; pH 4.0).**
(TIF)

Figure S3 **Summary of the NOE connectivities, J_HNα_ coupling constants (▾: J_HNα_<6 Hz), temperature coefficients and CαH chemical shift index for Anal3-Pro in SDS micelles.**
(TIF)

Figure S4 **Images of representative human RBCs illustrating the respective hemolytic effects of Anal 3-Pro and melittin: (** ***A*** **) No peptide treatment, (** ***B*** **) Anal 3-Pro and (** ***C*** **) melittin.**
(TIF)

Figure S5 **Scanning electron micrographs of untreated** ***C. albicans*** **(** ***A*** **) and cells treated for 4 h at 28°C with Anal 3-Pro (B) magainin II (C) or buforin II (D).**
(TIF)

Figure S6 **Electron micrographs of negatively stained SUVs composed of PC/choresterol (10∶1, w/w).** Panels show SUVs incubated with 4 µM peptide for 4 min. Bar = 100 nm (all) 1% UAC. (A) Control, (B) Anal 3-treated SUVs, (C) Anal 3-Pro-treated SUVs.
(TIF)

Figure S7 **DNA binding assay.** Gel-retardation experiments were performed by mixing 100 ng of the plasmid DNA (pBluscriptII SK+) with increasing the amount of peptide in 20 µl of binding buffer (5% glycerol, 10 mM Tris-HCl (pH 8.0), 1 mM EDTA, 1 mM DTT, 20 mM KCL and 50 µg/ml BSA). *A*, Anal 3; *B*, Anal 3-Pro; *C*, Buforin II; *D*, Tat. Lane 1, λ/Hind III size marker; 2, plasmid DNA alon; 3, 200 ng peptide; lane 4, 400 ng peptide; lane 5, 600 ng peptide; lane 6, 800 ng peptide; lane 7, 1 µg peptide; lane 8, 2 µg peptide; lane 9, 5 µg peptide; lane 10, 10 µg peptide; lane 11, 20 µg peptide.
(TIF)

Table S1 **^1^H Chemical Shifts (ppm) for Anal 3-Pro in SDS Micelles at 318K, pH 4.0.**
(DOCX)

Table S2 **Structural Statistics and Mean Pairwise rmsds for the 20 lowest energy Anal 3-Pro structures in SDS Micelles^a^.**
(DOCX)
