# Supplementary figures and images for: A Proline-Hinge Alters the Characteristics of the Amphipathic α-helical AMPs
Source: PLoS One. 2013 Jul 23;8(7):e67597. doi: 10.1371/journal.pone.0067597 (PMC3720801; doi:10.1371/journal.pone.0067597)

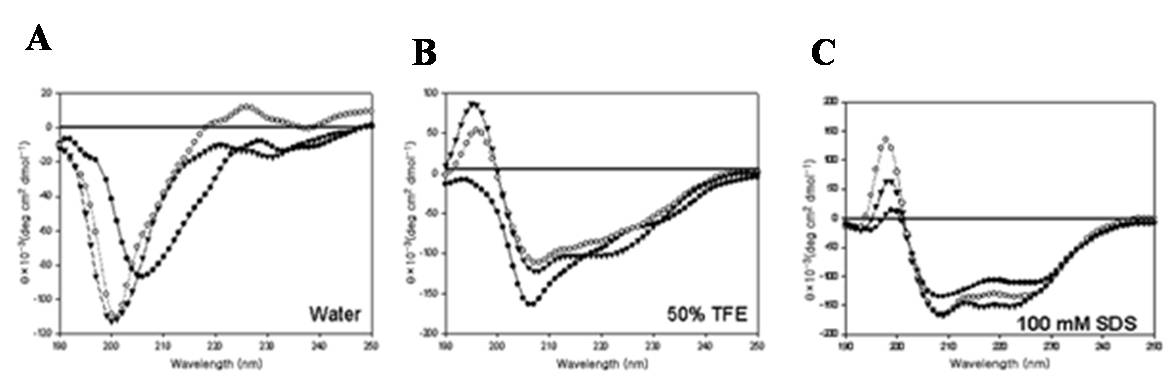

Supplement: Figure S1 — CD spectra for HP (2–20) (○), Anal 3 (▾) and Anal 3-Pro (•) in water (A) 50% (v/v) TFE (B) or 100 mM SDS micelles in 10 mM sodium phosphate buffer (pH 7.2) (C). In each case, the peptide concentration was 50 µM. (TIF) [file pone.0067597.s001.tif]

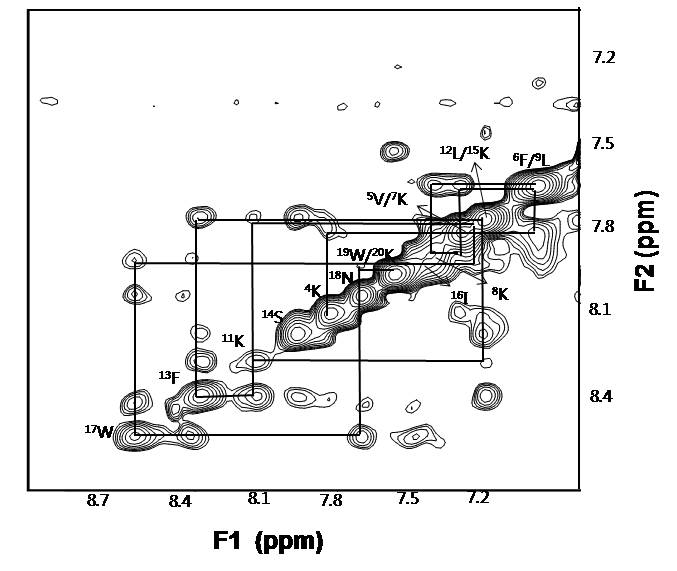

Supplement: Figure S2 — NH-NH region of the NOESY spectrum of Anal 3-Pro in 150 mM SDS micelles (mixing time, 350 ms; 318K; pH 4.0). (TIF) [file pone.0067597.s002.tif]

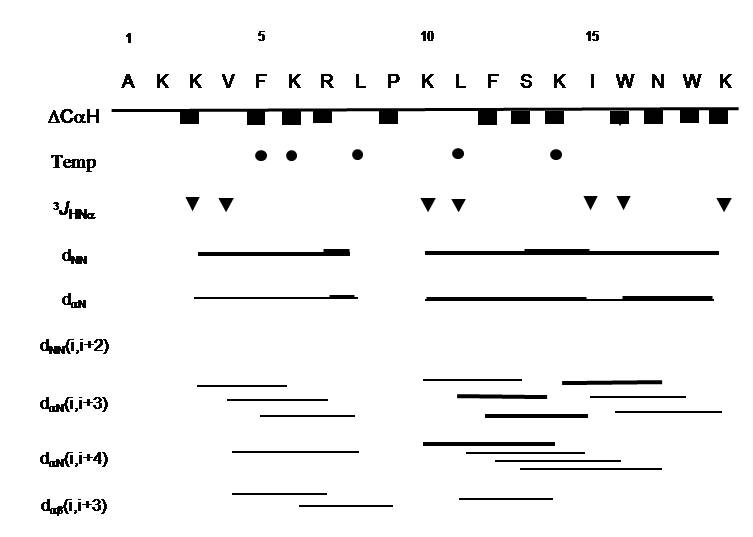

Supplement: Figure S3 — Summary of the NOE connectivities, JHNα coupling constants (▾: JHNα<6 Hz), temperature coefficients and CαH chemical shift index for Anal3-Pro in SDS micelles. (TIF) [file pone.0067597.s003.tif]

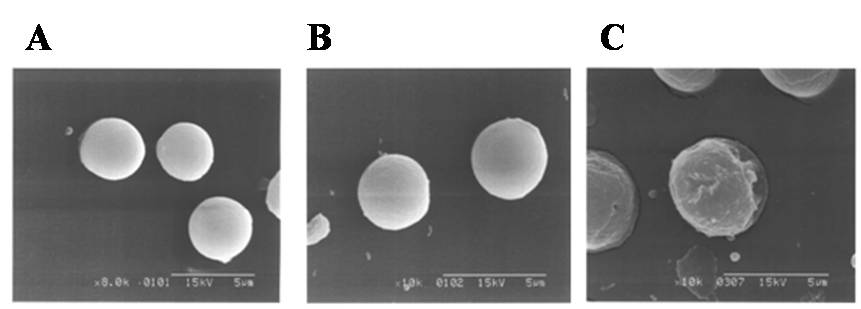

Supplement: Figure S4 — Images of representative human RBCs illustrating the respective hemolytic effects of Anal 3-Pro and melittin: ( A ) No peptide treatment, ( B ) Anal 3-Pro and ( C ) melittin. (TIF) [file pone.0067597.s004.tif]

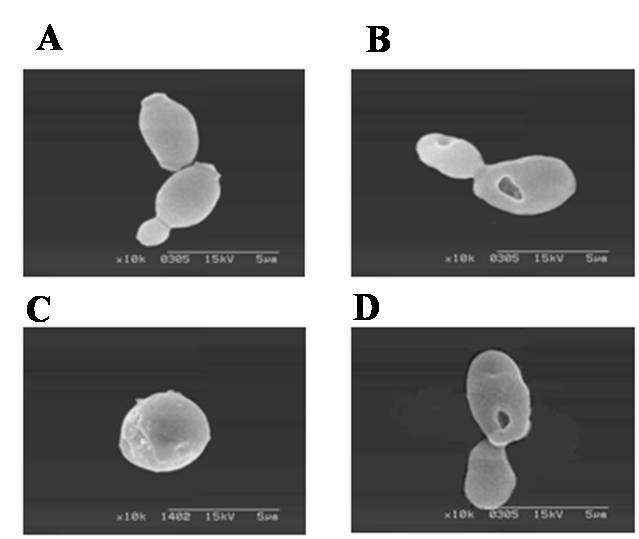

Supplement: Figure S5 — Scanning electron micrographs of untreated C. albicans ( A ) and cells treated for 4 h at 28°C with Anal 3-Pro (B) magainin II (C) or buforin II (D). (TIF) [file pone.0067597.s005.tif]

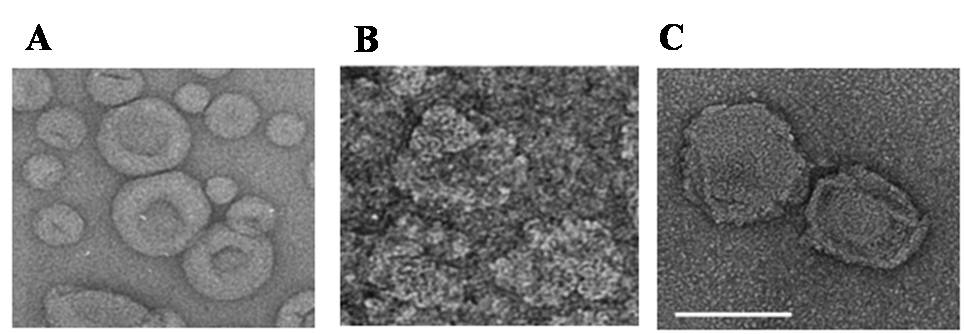

Supplement: Figure S6 — Electron micrographs of negatively stained SUVs composed of PC/choresterol (10∶1, w/w). Panels show SUVs incubated with 4 µM peptide for 4 min. Bar = 100 nm (all) 1% UAC. (A) Control, (B) Anal 3-treated SUVs, (C) Anal 3-Pro-treated SUVs. (TIF) [file pone.0067597.s006.tif]

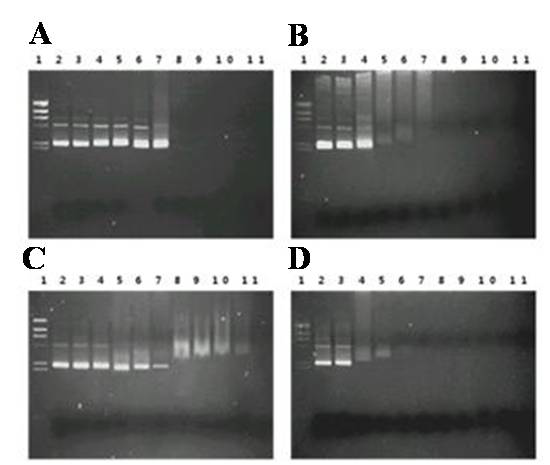

Supplement: Figure S7 — DNA binding assay. Gel-retardation experiments were performed by mixing 100 ng of the plasmid DNA (pBluscriptII SK+) with increasing the amount of peptide in 20 µl of binding buffer (5% glycerol, 10 mM Tris-HCl (pH 8.0), 1 mM EDTA, 1 mM DTT, 20 mM KCL and 50 µg/ml BSA). A, Anal 3; B, Anal 3-Pro; C, Buforin II; D, Tat. Lane 1, λ/Hind III size marker; 2, plasmid DNA alon; 3, 200 ng peptide; lane 4, 400 ng peptide; lane 5, 600 ng peptide; lane 6, 800 ng peptide; lane 7, 1 µg peptide; lane 8, 2 µg peptide; lane 9, 5 µg peptide; lane 10, 10 µg peptide; lane 11, 20 µg peptide. (TIF) [file pone.0067597.s007.tif]
