# Supplementary material for: A Proline-Hinge Alters the Characteristics of the Amphipathic α-helical AMPs
Source: PLoS One. 2013 Jul 23;8(7):e67597. doi: 10.1371/journal.pone.0067597 (PMC3720801; doi:10.1371/journal.pone.0067597)
Supplement: Table S1 — 1H Chemical Shifts (ppm) for Anal 3-Pro in SDS Micelles at 318K, pH 4.0. (DOCX) [file pone.0067597.s008.docx]

**Table S1.** ^1^H Chemical Shifts (ppm) for Anal 3-Pro in SDS Micelles at 318K, pH 4.0

| Residue | Chemical shift (ppm)^a^ | | | | ^3^*J*_H_  _NαH_ |
| --- | --- | --- | --- | --- | --- |
|  | NH | αH | βH | Others |  |
| Ala ^2^ |  |  |  |  |  |
| Lys ^3^ |  |  |  |  |  |
| Lys^4^ | 8.11 | 4.20 | 1.81 | rCH_2_ 1.44 | 3 |
| Val^5^ | 7.83 | 3.86 | 2.03 | rCH_3_ 0.82,0.64 | 4 |
| Phe^6^ | 7.67 | 4.42 | 3.22, 3.11 | 2,6H 7.32 |  |
| Lys^7^ | 7.83 | 4.16 | 1.90 | rCH_2_ 1.48 |  |
| Arg^8^ | 7.9 | 4.14 | 1.90 | rCH_2_ 1.75 |  |
| Leu^9^ | 7.67 | 4.19 | 1.88 | rH 1.45 /δCH_3_ 0.94,0.82 |  |
| Pro^10^ |  | 3.72 | 2.35 , 2.19 | rCH_2_1.83 / δCH_2_3.63 |  |
| Lys^11^ | 8.27 | 4.28 | 1.93 , 1.74 | rCH_2_ 1.51/ εCH_2_3.14 | 4  8 |
| Leu^12^ | 7.79 | 4.10 | 1.63 | rH 1.31 /δCH_3_ 0.89,0.81 | 3  6 |
| Phe^13^ | 8.38 | 4.28 | 3.14 | 2,6H 7.13 |  |
| Ser^14^ | 8.17 | 4.26 | 4.09 |  | 10 |
| Lys^15^ | 7.79 | 4.21 | 2.06 , 1.69 | rCH_2_ 1.55 | 5 |
| Ile^16^ | 7.93 | 3.89 | 1.97 | rCH_3_ 0.96 | 5 |
| Trp^17^ | 8.52 | 4.34 | 3.31 , 3.19 | 4H 7.47 / 2,6H 7.17 / 5H 6.96 |  |
| Asn^18^ | 8.05 | 4.49 | 2.86 | rNH_2_ 6.91 |  |
| Trp ^19^ | 7.97 | 4.49 | 3.48 , 3.39 | 4H7.56 / 2,6H 7.27 |  |
| Lys^20^ | 7.97 | 3.75 | 1.64 | rCH_2_1.16 / εNH_3_6.90 | 5 |

*^a^*Chemical shifts are relative to DSS (0 ppm)
