# Supplementary material for: A Proline-Hinge Alters the Characteristics of the Amphipathic α-helical AMPs
Source: PLoS One. 2013 Jul 23;8(7):e67597. doi: 10.1371/journal.pone.0067597 (PMC3720801; doi:10.1371/journal.pone.0067597)
Supplement: Table S2 — Structural Statistics and Mean Pairwise rmsds for the 20 lowest energy Anal 3-Pro structures in SDS Micellesa. (DOCX) [file pone.0067597.s009.docx]

**Table S2.** Structural Statistics and Mean Pairwise rmsds for the 20 lowest energy Anal 3‑Pro structures in SDS Micelles^a^

|  | Anal 3-Pro |
| --- | --- |
| Experimental distance restraints |  |
| Total | 126 |
| Sequential | 46 |
| Medium range | 24 |
| Intraresidue | 49 |
| Dihedral angle restraints | 7 |
| Rmsd from experimental restraints |  |
| NOE (Å) | 0.040±0.0015 |
| ф (deg) | 0.118±0.109 |
| Rmsd from convalent geometry |  |
| Bonds (Å) | 0.002±0.00007 |
| Angles (deg) | 0.518±0.01511 |
| Impropers (deg) | 0.339±0.02464 |
| Average energies (kcal mol^-1^) |  |
| E_tot_ | 46.35±2.33 |
| E_bonds_ | 1.89±0.12 |
| E_amgles_ | 28.10±1.65 |
| E_impropers_ | 3.59±0.53 |
| Rmsd from the mean structure |  |
| Backbone atoms of residues(10-19) | 0.77±0.25 |
| All heavy atoms of residues(10-19) | 1.46±0.37 |
